# Supplementary material for: The Molecular Medicine PhD program alumni perceptions of career preparedness
Source: PLoS One. 2022 Nov 17;17(11):e0275996. doi: 10.1371/journal.pone.0275996 (PMC9671420; doi:10.1371/journal.pone.0275996)
Supplement: S1 File — (PDF) [file pone.0275996.s001.pdf]

Supplemental File 1

Human Physiology and Disease. The purpose of this 10-week course is to give an introduction to the physiology of the major human organ systems, as well as selected associated pathophysiologies. The course provides a physiological basis for subsequent study and research in Molecular Medicine. The integration of clinical faculty into the course emphasizes the importance of bringing scientific knowledge to bear on clinical problems, a theme that is stressed throughout the MolMed curriculum. The course is mostly taught through self-directed learning, where study guide questions are provided in advance along with assigned reading in a human physiology textbook. During the class, the students lead the discussion of the study guide questions with help from the faculty facilitator. The course also begins to acquaint students with medical terminology.

Tools for Research. This 6-week course starts concurrent with the Physiology course, meeting daily in the afternoon, for a total of 15 hours/week. This course precedes the first lab rotation. There is both a didactic and lab component to this course. The goal is to review current laboratory methods essential to research in molecular medicine, and also to provide a hands-on laboratory experience to give all of our students, who come from various backgrounds, a common knowledge and practice base from which they can initiate their course work and lab. The didactic component includes an introduction to biostatistics through the use of Excel, Prism, and R Studio software, introduction to experimental design to promote rigor and reproducibility, methods to assess transcription, translation, miRNAs, and protein structure, and teaching about the uses and methods for epigenetics, next gen sequencing, site directed mutagenesis, protein expression and purification, antibodies, transfections and reporter gene assays, microscopy, and flow cytometry. The lab component includes tissue culture, RNA/DNA preparations, PCR, cDNA synthesis and cloning, cDNA expression in mammalian cells, protein analysis by ELISA and western blot, and protein purification. In addition, we use this course to introduce responsible conduct of research concepts about data integrity and reporting, a presentation and case-based discussion on sexual harassment and Title IX, and to present our interactive Lab Safety curriculum based on building a culture of safety.

Student Seminar Series (SSS). This 1-hour weekly seminar series is mandatory for all students in all years of the program and thus provides vertical integration of the students and fosters program identity. Students give seminars and address questions about their thesis or lab rotation research followed by constructive feedback on their slides and presentation style from two to three selected peers and the two faculty directors. Additional content in this course includes “Clinical Connections”, presentations by clinical co-mentors or other physicians to talk about the diseases they treat, and ongoing responsible conduct of research (RCR) training that consists of current real case studies followed by small group discussions led by Program trainers.

Cell Biology and Introduction to Pharmacology. This 6-week course includes a combination of interactive lectures and problem-based learning, journal clubs, and clinical correlations. Topics covered include cell structure and organelles, intracellular compartments and protein sorting, receptors, endocytosis, lipid rafts, the nucleus, cell communication and signal transduction, and mechanics of cell division. In addition we use this class to introduce principles of pharmacology including drug absorption, pharmacodynamics, pharmacokinetics, and drug discovery. The class stresses current methods and the design of experiments to test hypotheses. This class also uses journal clubs to teach content and presentation skills.

Metabolism. This 6-week course includes a combination of interactive lectures, research presentations, related journal club articles, and group projects with presentations. Topics covered include bioenergetics/oxidative phosphorylation, carbohydrate metabolism, lipid and lipoprotein metabolism, amino acid and nucleotide metabolism, vitamins, and integrative regulation of metabolism, diet, nutrition, and obesity. The class stresses methods and the design of experiments to test hypotheses.

Nucleic Acids, Gene Expression and Gene Regulation. This 6-week course includes a combination of interactive lectures, problem-based learning, and journal clubs. Topics covered include DNA structure, chromosome structure and confirmation, DNA replication and repair, RNA synthesis and processing, the

organization of eukaryotic genes, the genetic code, protein translation, epigenetics, and the regulation of gene expression. The didactic focus of this course is learning how to design experiments to test hypotheses and learning sufficient knowledge to be able to read and understand current literature in this field.

Mammalian Genetics, Genomics, and Bioinformatics. This 5-week course focuses on genetics, genomics, and bioinformatics taught through a combination of interactive lectures, problem-based learning, and a week-long group project. Topics covered include genetic variation, linkage studies, candidate gene and genome wide association studies, complex traits, linkage disequilibrium, pharmacogenetics, genome-wide expression studies, mouse models of human disease, and bioinformatics. Quantitative analysis and statistics are used throughout this course. The focus of this course is for students to be able to analyze various types of data, prepare graphics, and interpret the results. The course ends in a one-week bioinformatics project using R Studio software to analyze genome wide data.

Immunology. This 6-week course includes a reading program, interactive lectures, and problem-solving sessions. Clinical correlations relevant to weekly topics, are integrated into the material on a regular basis. Topics covered include biology and molecular biology of infectious agents, fundamentals of immunology, innate and adaptive responses to infection, immune effector mechanisms, and clinical aspects of immunobiology.

Principles in Clinical and Translational Research. This 10 week course in the second summer provides an introduction to the ethical, statistical, methodologic and informatics basis of clinical and translational research, and also includes a continuation of the pharmacology curriculum. Topics include the history of clinical and translational research, regulatory aspects of human and animal subjects research, clinical trials study design, conflicts of interest, human subjects recruitment, research and publication ethics, technology transfer, biobank construction and utilization, and clinical and research database construction and utilization. Each student writes about and presents a case study in bioethics. Students learn and utilize principles of biostatistics and clinical epidemiology relevant to clinical and translational research and gain mastery in statistical tools using problem based learning sets. This includes mastering competency in R (statistical computer language) for performing both simple and sophisticated statistical analyses. This course includes 9 hours of RCR small group case discussions led by Program Trainers, which are based on the chapters of the book “Scientific Integrity” by Francis Macrina.

Molecular Mechanisms of Human Disease/Grant Writing. This fall semester year 2 course meets 4 hours per week for 16 weeks, and it has two components: 1) a bedside to bench perspective of specific diseases and unmet needs; and 2) grant writing. The disease component presents one human disease in a bedside to bench perspective, with a different disease chosen each year aligning with student interests. Recent diseases covered in the past several years include type 2 diabetes, multiple sclerosis, colon cancer, muscular dystrophy, and lung cancer. The disease specific sessions start with presentations by clinicians, pathologists, clinical geneticists, and sometimes patients, who present cases and discuss current gaps in treatment and other unmet needs for that disease, and then from faculty conducting basic and translational research on each disease. Clinician-led field trips have included trips to cancer pathology labs, to robotic prostate surgery clinics, and to PET and MRI facilities. The last session of each disease block is for small group student presentations of specific aims/hypotheses that they developed for this disease and the methods and experiments they propose to use to address their aims. Parallel to the focus on these diseases, the rest of this course is focused on grant writing. This covers the mechanisms for NIH funding and peer review, the various sections of R01 style research grants, and writing assignments that culminate in writing the sections of a pilot grant proposal based on their thesis research plans. This proposal can then be expanded and used as the initial draft of the grant proposal that they submit for their qualifying exam. Finally, the class holds a mock study section in which all grants are reviewed by other students followed by scoring the grants using the NIH scoring system.

Clinical Experience Independent Study. One of the most unique features of the MolMed Training Program is the integration of clinicians into PhD training. The Thesis Advisor and the student, with the

approval of the Clinical Experience Course Director, select a Clinical Co-Mentor to advise the student and serve on the student's thesis/qualifying exam committee. The role of the Clinical Co-Mentor on the thesis committee is to help the trainee focus on translational and clinical perspectives of their research, which may lead to direct collaborations or connections with other clinicians who may get involved with the project. The Clinical Co-Mentor works closely with the student to create an individualized proposal for activities during the Clinical Experience course. The proposal is subject to approval by the Clinical Experience Course Director before the student begins this independent study experience. This course requires 30 logged hours of clinical activities, excluding Grand Rounds or seminars. A variety of clinical activities and attending physicians must be proposed and must include patient interventional and diagnostic experiences. The student must submit the completed and signed log along with a narrative summary of their Clinical Experience to the Course Director. These and other clinical experiences often continue throughout the student's Ph.D. training. In addition, this exposure to the clinic, can spark interest in careers such as clinical research project management, which is a career within the biomedical workforce arena that a few of our graduates have moved into. We have observed several long-term interactions develop between the student and their Clinical Co-Mentors, with students continuing clinical activities throughout their PhD research period

Elective Courses. Students may enroll in any graduate level course from the Case Western Reserve University (CWRU) catalog, based on the recommendation of their thesis advisor and thesis committee. Often, students will take additional electives in the area of their research in order to acquire more expertise and knowledge. In addition, courses and other educational activities are available at the Cleveland Clinic and CWRU in teaching, management, and entrepreneurial and professional skills that can help prepare students for diverse careers.

Other Training Requirements and Student Oversight

Lab Rotations. Students spend their first year in two to four 8 to 12-week rotations in laboratories of qualified trainers. The Program Director serves as the advisor to these students and councils them on finding a good match for their interest and lab style preferences. At the end of each rotation, the student writes a 2-3 page Rotation Report including a description of the problem being studied, the hypothesis, experimental methods, results and interpretation, future directions, and references. Students orally present their work from one of their rotations in the Student Seminar Series.

Thesis Advisor and Committee. At the beginning of their second year, the student decides on their Thesis Advisor, which is subject to sufficient funding and approval by the advisor's Department Chair and the Program Director. The student works with the Thesis Advisor to determine the thesis topic and the members of the Thesis Committee, which includes the clinical co-mentor. The Thesis Committee meets twice per year. To ensure timeliness, students are told that it is better to schedule the meeting on time even if one committee member is not available. Students prepare a 2 page summary of research progress prior to each meeting. The agenda for the meeting is provided in the Program handbook. The committee chair fills out an evaluation form of progress towards degree along with a narrative about the student's strengths and targeted areas for improvements. After every thesis committee meeting, the student schedules a meeting with the Program Director, and provides their 2-page research summary and the committee evaluation form. This meeting is another chance for bidirectional feedback, where the Director can assess the science, the progress towards degree, and reinforce the comments of the thesis committee. The student is also asked about their professional relationship with their advisor, the lab environment, Program activities, and any issues regarding their well-being.

Qualifying Exam. Students take a qualifying exam in year 2, which includes a grant proposal based on their thesis research, without help from their thesis advisor aside from the Aims page, a short oral presentation of the proposal, and an oral defense of the proposal and other knowledge from the core curriculum. The Exam committee is the thesis committee plus a member of the executive committee (if not already on the committee), who chairs the exam. The outcomes are Pass or Provisional Pass, which requires a revised proposal based on written comments from the Exam chair.

Individual Development Plan and Annual Student Review. Every year the students completes an online individual development plan (IDP) to answer questions about goals, accomplishments, and networking with other faculty. Then there is an iterative online written response and evaluations from the thesis advisor followed by a scheduled face to face discussion with the student and their thesis advisor. Every year in the fall, the Executive Committee performs an annual review of each trainee in the program. The following material is available to the reviewers: the student's updated CV, thesis committee reports, academic transcript, rotation reports, and IDP. The committee then decides on an assessment that is sent in a letter to the student and their thesis advisor, stating whether the student is exceeding, meeting, or not meeting our expectations towards progress to degree, based on a rubric according the student's year in the program. We find that this yearly formal review gives both our trainees and advisors a realistic assessment of the student's progress and whether there are any concerns that need to be addressed.

Publication Requirement. The program has a flexible minimum publication requirement for graduation, with one first (or co-first) author paper accepted and an additional first (or co-first) author paper submitted. However, the student can petition the Program's executive committee for waivers, so as not to be subject to long delays based on the mentor's choice of journal and overbearing revisions requested by journal reviewers. The thesis committee ultimately decides when the student has met the research threshold for a body of work worthwhile of granting the PhD.

Professional and Career Development Opportunities.

Providing information about a variety of careers. The Research Education and Training Center (RETC) at the Cleveland Clinic Lerner Research Institute provides an extensive array of professional and career development seminars and panel discussions every year given by internal and external speakers from a variety of different positions, which we highly encourage our students to attend. The following is the list of titles of these seminars and panel discussions offered in the past two years: Setting Your Career Goals; Resources Available to Lerner Trainees; Professional Communication; Getting the Most from your Mentor; Bench to Boardroom to Bedside; Taking Care of Yourself; Professionalism; Introduction to Clinical Research; Conflict Resolution; Starting Your Own Lab; International Trainee Resources; Bioinformatics; Biostatistics; Virtual Networking; Grant Writing; Posters; Graphics for Presentation; Writing Papers; My Thoughts on Science Communication; The Ins and Outs of Thesis Committees; Medical Affairs, a Spotlight on the Medical Science Liaison Role; A Case Study: From Grad School to Business Career Without a Postdoc or MBA; Marketing Yourself for Non-academic Careers; From Postdoc to Senior Scientist: My Journey from Academia to Industry; Stress Management & Resources During the Pandemic; and Careers for Scientists in Patent Law. Additional career development seminars and workshops are available at the CWRU School of Medicine (SOM).

Professional Skills, and Networking Development. Our curriculum and other training activities go a long way in training the students in many skills needed to attain positions in various sectors of the biomedical workforce. For example, skills in problem solving, creative thinking, research design for rigorous and reproducible results, statistics, verbal, and written communication are acquired throughout a student's tenure in our Program. These skills prepare our trainees for many diverse jobs in the biomedical workforce. The independent clinical experience also leads to new skills and appreciation for clinical research, which probably led some of our graduates obtain positions as clinical research project managers. Although our program has no teaching requirements, we have several teaching opportunities for our students. First, several of the more senior students have given seminars within the core courses. Second, every year current students serve as tutors for the Gene Regulation course, the Immunology course, and the biostatistics/epidemiology component of the Principals of Clinical and Translational Research course. Third, the CWRU SOM ExTEnD course offers formal teacher training and teaching experience. We also have a peer mentoring program where year 2 and/or 3 students assist year 1 students entering the training program, giving our students the opportunity to learn skills in mentoring, which can also be useful for various positions in the workforce. Our trainees are encouraged to attend scientific meetings where they present posters or oral presentations, giving them both learning and networking opportunities. CWRU SOM offers an internship program called Enhancing Research and Industry Career Horizons (EnRICH). A student can spend time for a paid or non-paid work

experience that is beneficial to both the employer and student. The duration of the experience is flexible where the mentor and student agree on the duration of the work experience and to an hourly and weekly work schedule. During the experience, students will clarify career goals as she realizes the results of applied skills in a non-academic career, identifies ways to adapt skills for a variety of occupations, gains broader perspectives of careers that require her skills and talents, learns the business side of science and technology, and develops personal and interpersonal skills for relationship building to broaden professional networks. Recently, two of our students did internships, one at clinical genetics biotech, and the other was awarded a Science and Technology Policy Fellowship at the National Academies of Science, Engineering, and Medicine in Washington, D.C. An additional external experience that one of our students participated in was the Programming for Biology course at Cold Spring Harbor Laboratories. The 16-day course included lodging, meals and course materials. The student stated that "I would not have had the abilities to do analysis needed for the paper that I am currently assembling if it were not for this course."
