# Supplementary material for: The Molecular Medicine PhD program alumni perceptions of career preparedness
Source: PLoS One. 2022 Nov 17;17(11):e0275996. doi: 10.1371/journal.pone.0275996 (PMC9671420; doi:10.1371/journal.pone.0275996)
Supplement: S2 File — (PDF) [file pone.0275996.s002.pdf]

Supplemental File 2

Molecular Medicine Post-Graduation Program Review and Outcomes Survey

Responses will be confidential and anonymous to the program leadership. Data collected from this survey will be used to help improve the program and provide required information to the NIH. We plan to continuously evaluate our alumni and their experience as students, and we will be sending this survey periodically. We appreciate your participation and feedback.

General Information

Month and year of program entrance (ex July 2013)*

Month and year of Graduation (ex May 2017)*

Employment Post Degree

Please list all employment with corresponding information since graduation

Job 1 after graduation

How many year(s) were you in this position?

-- None --

1
2
3
4
5
6+

Employment Sector

-- None --

Biotech/Pharma
Education College/University
Education K-12 or other non-college
Government
Hospital
Other Business or Industry
Postdoctoral Fellow
Self-employment
Other

Job 2 after graduation

How many year(s) were you in this position?

-- None --

1
2
3
4
5
6+

Employment Sector

-- None --

Biotech/Pharma
Education College/University
Education K-12 or other non-college
Government
Hospital
Other Business or Industry
Postdoctoral Fellow
Self-employment
Other

Current Job*

How many year(s) have you been in this position?

-- None --

1
2
3
4
5
6+

Employment Sector

-- None --

Biotech/Pharma
Education College/University
Education K-12 or other non-college
Government
Hospital
Other Business or Industry
Postdoctoral Fellow
Self-employment
Other

Other jobs held after graduation that could not be listed above with number of years worked and sector.

How many original research papers have you published in positions post-graduation?

-- None --

0
1
2-4
5-9
10

Post-graduation have you received research grants from:

	Yes	No
US Fed. Government	○	○
Other Sources/Private Foundation	○	○

Important Elements of Education & Factors of Landing First Job

For doctoral students interested in your career field (broadly defined), how important are the following elements of your Molecular Medicine PhD training?

	Not important at all	Not very important	Important	Very Important
Experience gained through Molecular Medicine PhD classroom curriculum	○	○	○	○
Experience working collaboratively with students and other lab personnel	○	○	○	○
Experience working with your thesis advisor and committee	○	○	○	○
Exposure to careers outside of academic scholarship/research	○	○	○	○
Practice of research methods in your field	○	○	○	○
Presentation of work at a professional conference and/or seminar	○	○	○	○

How prepared did you feel entering your career field based on the Molecular Medicine PhD classroom curriculum?

-- None --

Not prepared at all
Not very prepared
Prepared
Very prepared

Please list classes/topics/curriculum that contributed to your preparedness

Please list suggestions of classes/topics/curriculum that should be added.

Which of the following were significant factors in helping you to land your current position:
(Mark all that apply)

- ☐ **Career readiness programs**
- ☐ **Clinical Experience/focus on translational research**
- ☐ **Connections and networking with CWRU/ CCF program alumni**
- ☐ **Perceived quality of my academic work**
- ☐ **Reputation of Case Western Reserve University and Cleveland Clinic**
- ☐ **Reputation of Molecular Medicine Program**
- ☐ **Support and activity of thesis advisor, thesis committee, other CCF/CWRU faculty, or program administration**
- ☐ **Other significant factors**

Competencies and Preparation for First Position

Please indicate your level of agreement with the following statements.

The Molecular Medicine PhD program content supported my research and or professional goals.

- ○ **Strongly Disagree**
- ○ **Disagree**
- ○ **Agree**
- ○ **Strongly agree**

Please provide a short supporting reason for your selection.

How important are each of the following competencies for doctoral students entering your field?

	Not important at all	Not very important	Important	Very important
Academic and professional writing	○	○	○	○
Bench-based wet lab research	○	○	○	○
Ethics and Integrity	○	○	○	○
Genetics/Bioinformatics	○	○	○	○
Human Physiology and Disease	○	○	○	○
Medical knowledge including the Clinical Experience course	○	○	○	○
Molecular/Cellular Biology	○	○	○	○
Statistics/Epidemiology	○	○	○	○
Other Competencies	○	○	○	○

List other competencies*

How satisfied were you with the Molecular Medicine PhD curriculum and training in these competencies?

	Very dissatisfied	Dissatisfied	Satisfied	Very satisfied
Academic and professional writing	○	○	○	○
Bench-based wet lab research	○	○	○	○
Ethics and Integrity	○	○	○	○
Genetics/Bioinformatics	○	○	○	○
Human Physiology and Disease	○	○	○	○
Medical knowledge including the Clinical Experience course	○	○	○	○
Molecular/Cellular Biology	○	○	○	○
Statistics/Epidemiology	○	○	○	○
Other Competencies	○	○	○	○

List other competencies*

Please provide at least one suggestion on how the Molecular Medicine PhD curriculum could better align with the listed competencies.

Satisfaction with Current Position

How satisfied are you with your current position?

The work itself is interesting, rewarding and/or meaningful to you.

- ◯ Very dissatisfied
- ◯ Dissatisfied
- ◯ Satisfied
- ◯ Very Satisfied

Would alumni do it again?

Given the perspective you have gained since completing the Molecular Medicine PhD program, if you could start again, what would you do? Would you again pursue a doctoral degree?

- ◯ Definitely not
- ◯ Probably not
- ◯ Probably
- ◯ Definitely

Please list why you would not pursue a doctoral degree again.

What advice would you give to an incoming student?

Please provide any additional information you would like to share with Molecular Medicine PhD program.
