# Supplementary material for: The Molecular Medicine PhD program alumni perceptions of career preparedness
Source: PLoS One. 2022 Nov 17;17(11):e0275996. doi: 10.1371/journal.pone.0275996 (PMC9671420; doi:10.1371/journal.pone.0275996)
Supplement: S3 File — (PDF) [file pone.0275996.s003.pdf]

Supplemental File 3

Hello!

Hope you are doing well.

The Molecular Medicine program is collecting data to help improve the program and provide required information to the NIH. We plan to continuously evaluate our alumni and their experience as students, and we will continue to send this survey periodically.

Responses will be confidential and anonymous to the program leadership.

We appreciate your voluntary participation and feedback, please see the link below and complete the survey by [enter date].

http://survey.clevelandclinic.org/mmedalumni

All our best,

Molecular Medicine Admin Team

For questions about this survey or to opt out of future surveys, please contact Jonathan Smith at smithj4@ccf.org

As this survey qualifies as human subjects' research, it is subject to IRB approval. For questions about this please contact the Cleveland Clinic IRB at irb@ccf.org protocol 07-542.
